# Stress, Anxiety and Depression Prevalence among Greek University Students during COVID-19 Pandemic: A Two-Year Survey

**DOI:** 10.3390/jcm11154263

**Published:** 2022-07-22

**Authors:** Dimitrios Kavvadas, Asimoula Kavvada, Sofia Karachrysafi, Vasileios Papaliagkas, Stavros Cheristanidis, Maria Chatzidimitriou, Theodora Papamitsou

**Affiliations:** 1 Post-Graduate Program “Health and Environmental Factors”, School of Medicine, Faculty of Health, Aristotle University of Thessaloniki, 54124 Thessaloniki, Greece; akavvada@auth.gr (A.K.); sofia_karachrysafi@outlook.com (S.K.); stavrcher@auth.gr (S.C.); thpapami@auth.gr (T.P.); 2 Laboratory of Histology and Embryology, School of Medicine, Faculty of Health, Aristotle University of Thessaloniki, 54124 Thessaloniki, Greece; 3 Department of Biomedical Sciences, School of Health Sciences, International Hellenic University, 57400 Thessaloniki, Greece; vpapal@auth.gr (V.P.); mchatzid952@gmail.com (M.C.); 4 Laboratory of Atmospheric Physics, Department of Physics, Aristotle University of Thessaloniki, University Campus, 54124 Thessaloniki, Greece

**Keywords:** mental health, college, students, DASS21, pandemic, gender

## Abstract

Background: The negative effect of COVID-19 pandemic on college students’ mental health is well-demonstrated. The aim of this study is to assess the impact of the pandemic on the students of Aristotle University of Thessaloniki (Northern Greece), in terms of stress, anxiety, and depression, and to analyze the probable correlation of various social and phycological factors. Methods: The survey was conducted in the form of a questionnaire, which was first distributed in November 2020 and then re-launched in November 2021. The evaluation was carried out through the DASS21 screening tool. Associations regarding participants’ characteristics and the three variables (stress, anxiety, and depression) were investigated with Pearson’s chi-squared (Χ^2^) test. Results: The first-year results (November 2020) revealed severe prevalence of stress, anxiety, and depression (37.4%, 27.2% and 47% respectively). The second-year results (November 2021) revealed a significant augmentation in all three variables, mainly for the extreme severe scales (47.3%, 41.1% and 55% respectively). Participants who were receiving psychiatric treatment exhibited higher levels of stress, anxiety, and depression, especially during the second year of the pandemic (*p*-Value < 0.00001). Female students’ mental health was at higher risk, as elevated prevalence of negative symptoms was observed (*p*-Value < 0.00001). Conclusions: The community of Aristotle University of Thessaloniki has been greatly affected during the last 2 years. The inherent risks of the confinement measures on students’ well-being and mental health are undeniable. Recurrent annual psychological evaluation in universities and colleges is strongly advised.

## 1. Introduction

An unknown etiology outbreak of pneumonia turned into the pandemic of COVID-19 (Coronavirus disease-19) disease and spread rapidly throughout the planet [1,2]. The mortality was unexpectedly high, leading countries to take drastic measures [3].

Several studies focused on the correlation among demographic characteristics and psychological deterioration during the pandemic [4,5,6]. Remarkable efforts have been made on the evaluation of psychological and mental effects on university students. Evidence suggest an increase in stress, anxiety, and depression levels in several countries. In China, according to Ma et al., the probability of developing acute stress, depression, and anxiety disorder was 34.9%, 21.1%, and 11.0% respectively, based on a sample of 746,217 students during November 2020. In the United States, in a similar study conducted during 2020, 195 students were evaluated and 71% of the sample revealed increased stress and anxiety during the pandemic, in comparison to the pre-COVID period [7,8,9]. Post-traumatic stress disorder, accompanied by sleep disorders, has also been observed. These findings confirm that intense and constant concern can lead to a severe reduction of the academic performance and to psychological health distress. For instance, according to Son et al., over 90% of the sample of university students expressed concerns for their relatives’ well-being and 89% had difficulties to concentrate. Almost 86% reported sleep disorders and deterioration of their social life. Moreover, 82% observed decreased performance in their academic courses. Similar reports were recorded by Tang et al., during the first year of the pandemic, on 2485 students from six Chinese universities [7,10]. An aggravating factor in the above is the loss of their jobs or the alteration to their employment routine [11]. Several studies suggest that younger age, poor health, extensive exposure to computer screens, and the constant fear for SARS-CoV-2 infection, pose significant risk factors for the students’ mental health during the pandemic. There is a worldwide indication that female gender should be included in the risk factors. Chang et al. observed that female students were more likely to suffer from anxiety and depression during the COVID-19 period [9]. Browning et al. collected data from seven United States Universities during the first months of the pandemic. Their findings suggest that female gender is hampered by the already deteriorating mental health of American students during the pandemic [12]. Similar observations were made in a survey conducted in France, including 69,054 students during the first lockdown period of 2020 [13]. Other risk factors are financial hardship, distance learning, and uncertainty about the academic future due to the ongoing preventive measures and quarantine [14].

A comparative study revealed severe stress management imbalance during the pandemic, compared to 2018 [15]. Similarly to other studies, it was noticed that female students and students with disabilities and different sexual orientation respondents faced significant difficulties and their mental health was aggravated during the pandemic [15,16].

Studies were also carried out in Greece during the first year of the pandemic, presenting similar results. Kaparounaki et al. conducted a study on student mental health during the first months of the pandemic in Greece (April 2020). Among the 1000 participating students, increased stress and depression levels (42.5% and 74.3% respectively) were observed [17]. Patsali et al. collected data from April to May 2020. A total of 1104 female Greek students and 431 male Greek students participated in the study. The results showed that more than 65% of the respondents reported a significant increase in stress due to quarantine. Severe depression and feelings of despair were also reported. Female gender appeared as a potential risk factor of depression [18]. Moreover, a study on 1060 students (mostly females) from several Greek universities during the first months of the pandemic [19] was performed through the Depression Anxiety Stress Scale (DASS-21). Moderate to extreme severe depression was observed in 35.6% of the students, while 31.8% and 19.7% reported moderate to extreme severe stress and anxiety, respectively. The prevalence of negative emotions was particularly high compared to similar rates on Chinese students, but similar to Mediterranean countries like Italy and Spain [19].

The research hypothesis expected increased levels of stress, anxiety, and depression in the second distribution of the survey. In addition, it was assumed that demographic and individual characteristics of the participants (such as gender, cohabitation status, vaccination status, psychological or psychiatric treatment) were significantly correlated with the different levels of stress, anxiety, and depression. The hypothesis was derived from recent global data with evidence of continuous deterioration of university students’ mental health during the pandemic [7,8,9,10,14].

## 2. Materials and Methods

### 2.1. Study Design

The study was conducted through an anonymous questionnaire, which assessed the psychological, social data of the participants and included the Depression Anxiety Stress Scale (DASS21). The survey was launched twice. The questionnaire was the same for both distributions.

The aim of this research was to assess the general mental health of the Aristotle University of Thessaloniki (AUTh) students during COVID-19 “peak” periods, in Greece. For this purpose, DASS21 screening test was used in order to evaluate stress, anxiety, and depression levels. Furthermore, the impact of physical, social, and phycological factors and their probable correlation to the students’ current mental state were analyzed. According to the literature, a further gender analysis was considered to be essential.

### 2.2. Population and Samples

**Figure 1:**
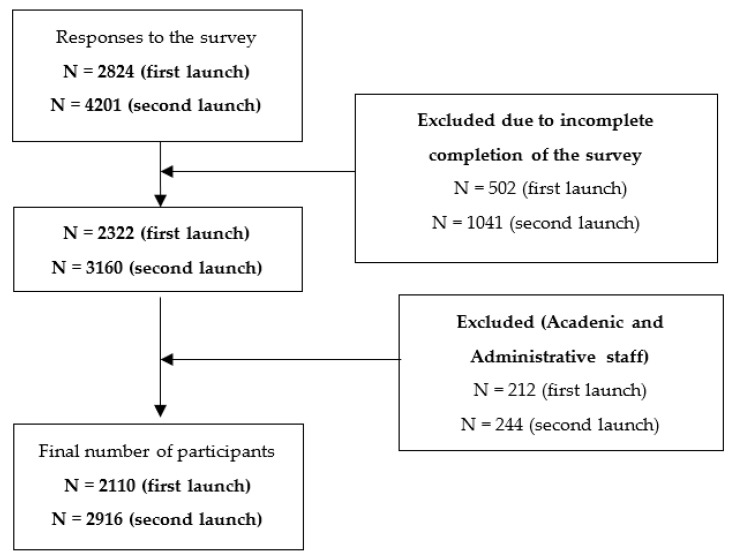
Participants’ flow chart with inclusion and exclusion criteria.

The study sample included students (BSc, MSc, PhD) of the university. The research was conducted at two time intervals. The first period of the survey took place in November 2020 (first major peak of the pandemic) and included 2322 participants, while the second was in November 2021 (second peak) with 3160 participations. Based on the official registration of the AUTh, as of November 2021 there were 51,577 active students [20]. Therefore, the collected students’ samples represent approximately 5–6% of the students’ population (2110 and 2916 for the first and the second year respectively) (Figure 1). The rest participants were excluded as they represented the academic and administrative staff of the university. It should be noted that more than 1500 respondents’ entries were also excluded from the surveys due to incomplete completion of the questionnaire (Figure 1). The percentage of female active students was approximately 54% and of the male active students was 46%.

### 2.3. Questionnaire Platform and Approvals

The hosting platform was the LimeSurvey AUTH (Aristotle University of Thessaloniki) under the supervision of the certified questionnaires authority of Aristotle University). Due to the legislation on personal data protection (GDPR), both the AUTh bioethics committee (Bioethics Committee No. 1.254/20-10-20) as well as the AUTh Data Protection office, granted permission. The LimeSurvey AUTH platform gathered the responses and access was granted only to the head professor of the project (author T.P.) via a personalized link, which created a secure authorized profile.

### 2.4. Distribution and Content of the Questionnaire

The questionnaire was available from 15 November 2020 (08.00 A.M. Greece time) to 30 November 2020 (08.00 A.M.) (first launch) and from 15 November 2021 (08.00 A.M.) to 30 November 2021 (08.00 A.M.) (second launch). The survey was conducted in the form of an online questionnaire, which was distributed to participants via their institutional e-mail (“name”@auth.gr). All of the questions were closed-type questions and it was obligatory for participants to choose an answer, once they agreed to participate, in order to complete the survey.

The questionnaire was divided into three thematic sets (Table 1):

Τhe first set was about the basic information and participant’s profile, psychological evaluation, and experiences in relation to COVID-19 [16]. The aim was to extract information related to the basic characteristics of the respondents (questions 1–6) and their familiarization with COVID-19 (questions 7–10). Moreover, there was a short evaluation of their psychological state (questions 12–14) and their social well-being (questions 16–18).

The second section was about the university status (questions 19–21)

Both sets 1 and 2 were composed of well-established questions, similar to other published studies [16].

The third and most significant set of questions (questions 22–42) assessed the levels of anxiety, stress, and depression, as well as their physical manifestations, through the DASS21 [16,19].

The DASS21 (Depression, Anxiety, and Stress Scale) was introduced in 1995 by Lovibond and Lovibond [21]. It consists of three self-report scales designed for screening of depression, anxiety, and stress [16,19]. In 1998, a final version of the DASS that consisted of 21-item (DASS21) was described [22]. Each of the three DASS21 scales contain seven elements, divided into subscales with similar content. The Depression Scale assesses discomfort, despair, life devaluation, self-devaluation, lack of interest/engagement, and inaction. The stress scale assesses autonomic arousal, signs of stress through skeletal muscle movements, stress-induced anxiety, and the subjective experience of anxiety. The stress scale is sensitive to chronic non-specific stimulation. This scale evaluates the difficulty of relaxation, nervous agitation and upset/agitation, the case of an irritable/hyper-reactive characters, and impatience. Scores for depression, anxiety, and stress are calculated by summing the scores for the relevant data [21,22]. The DASS21 rating scale is used internationally to assess stress, anxiety, and depression levels. It is a recognized and accepted tool by psychologists and psychiatrists with a very good internal consistency [22]. It is, therefore, a valid Likert-4 scale (0. Not at all, 1. A little, 2. much, 3. Too much), which calculates the negative emotional states experienced by the participants during the period of time that the survey is available. The Greek version of the DASS-21 scale, based on Greek sources and official translations, was described by Lyrakos et al. [23]. This particular version was used in this survey. The results can be either normal, mild, moderate, severe, or extremely severe. For stress, a normal score is 0–7, 8–9 for mild stress, 10–12 for moderate, 13–16 for severe, and above 17 for extreme severe stress [21]. Similarly, 0–3 is the normal score for anxiety, 4–5 is the prevalence of the mild anxiety, 6–7 moderate, 8–9 severe and above 10 is the extreme severe anxiety [21]. Finally, a score of 0–4 is normal for depression, 5–6 is mild, 7–10 is moderate, 11–13 is severe, and above 14 is the score for extreme severe depression [21]. The scores for depression, anxiety, and stress are calculated by summing the scores for the relevant items [21].

The above questionnaire was distributed for the second time on November 2021. The second launch included the same questions with two extra questions (vaccine coverage and concerns for an impending lockdown).

During the 2-year study, all personal data protection measures were obtained for the participants, who were informed that they had the right to terminate their participation at any time. They were also informed about the purpose of the research, the population target of the university, and the DASS21 evaluation scale. It was stated to them that any processing of personal data would be done in accordance with the General Regulation of Personal Data Protection, taking the appropriate technical and organizational measures. Personal data were kept only for the period required for the lawful purposes for which they were collected ensuring their safe destruction, when the legally abovementioned period had elapsed or the purpose of their processing ceased to exist. Finally, they were informed that for the purposes of the investigation it was not required to verify their identity by those responsible for processing the data, with the result that the latter were not obliged to obtain or retain or process additional information to verify the identity of each participant. Consequently, the following rights did not exist: (a) the right of access to personal data, (b) the right of correction, (c) the right of deletion, (d) the right of restriction of processing, and (e) the right of data portability in accordance with the General Regulation Personal Data Protection. Contact details were provided for anyone seeking more information and clarifications.

### 2.5. Statistical Analysis

The Cronbach “alpha” factor was excellent in both years. More specifically, it was estimated at 0.946 for the DASS21 launched in 2020 and 0.954 for the DASS21 launched in 2021. As mentioned, the DASS-21 is based on a multi-dimensional and not a categorical perception of psychological distress [21,22]. The hypothesis on which the development of DASS21 was based (and which was confirmed by research data) is that the differences between depression, anxiety, and stress experienced by normal individuals and clinical populations, are gradually different [21]. The demographic characteristics of the participants and the answers to the first set of questions (psychological assessment, COVID-19, academic capacity) were studied with Pearson’s chi-square test. Because of the large set of categorical variables, the multiple correspondence (correlations) analysis was performed for the second questionnaire (November, 2021) [24,25,26,27,28]. The aim was an in-depth psychological evaluation of the students after 2 consecutive years of the ongoing pandemic, during the second and largest peak of COVID-19 outbreak in Greece. The results of the 21 questions through which the score of anxiety, stress, and depression was obtained, are summarized within the multiple correspondence analysis in in three grades (Normal, Mild to Sever, Extreme Severe). The gradation was delineated based on the quadrants Q1 and Q3 of a continuous distribution of samples with a value range of 0–21, which consist of the sum of the DASS21 scores [29]. The multiple correspondence processing leads to the construction of the Burt tables, which are multiple coincidence tables. These tables were produced by the intersection of the classes of each variable [30]. The purpose of the multiple correspondence analysis is the calculation of the coordinates of the rows and columns on the factorial axes that are formed during the analysis of the data, in order to interpret the extracted information [24,25,26,27,28]. In this case the columns represented the levels of stress, anxiety, and depression, while the rows were the responses to the basic information received by the first part of the survey (demographic, psychological etc.) The statistical processing of the results was performed with the program SPSS version 24.0 (IBM, SPSS Inc., Chicago, IL, USA), Microsoft Excel (2019) version 16.43, and the the Méthodes d ‘Analyses des Données (MAD) software [31].

## 3. Results

### 3.1. Two-Year Demographic Data

Female participants outnumbered males in both years (70% and 73%, first and second launch respectively). The cohabitation status changed in the second year, with a higher percentage stating to dwell in their houses without roommates (31%) or with one person (25%) (chi-square 281.2, *p*-value < 0.00001), compared to 2020 (15% and 21% respectively). Significant difference was also observed in the work status, with more employees reporting changes in their job routine during the first year (27.5%) (chi-square 23.8, *p*-value < 0.00001). During the second launch (November 2021), the majority of the participants reported to know an acquaintance who was diagnosed with COVID-19 (94.4%). This rate was significantly increased in comparison to the first launch (chi-square 81.5, *p*-value < 0.00001). Meanwhile, the cases of acquaintances who were diagnosed positive and were seriously ill or died were also significantly increased in 2021 (21%) (chi-square 99.0, *p*-value < 0.00001). Psychiatric care (chi-square 78.3, *p*-value < 0.00001) and psychotropic drugs intake (chi-square 14.3, *p*-value 0.00015) were also increased during the second year of the pandemic (14.5% and 3.9% respectively). Also, compared to the first year, the respondents felt that the relationship between people confined in the same house was significantly deteriorated (50%) (chi-square 12.4, *p*-value 0.00042).

The majority of students who had not been vaccinated by the time of the second launch were undergraduate and master students (25% and 19% respectively).

A statistically significant difference was observed regarding gender and the fear of an impending lockdown, due to the raised numbers of COVID-19 cases (*p*-value < 0.00001). The majority of females (60%) were more concerned compared to male participants (50%) (Supplementary Material; Tables S1–S14).

### 3.2. DASS-21 Results

The results of the DASS21 surveys are presented below (Table 2). In 2020, moderate to severe stress and extreme anxiety was observed in high rates. In the same year, 60% of the depression prevalence rates in students were distributed to the scales from mild (13%) to extremely severe (16.1%). In 2021, a significant increase was observed in all extreme severe scales (Table 2).

In the second launch of the survey (November 2021), the extreme scale values for both female and male students were almost doubled (Table 3 and Table 4).

A further study was performed on those who knew someone diagnosed with COVID-19 during the 2 years. The percentage of those who were familiar to someone who died of COVID-19 during the second year of the pandemic was doubled, in comparison to the first year (*p*-Value 0.0002).

In order for an in-depth analysis to be performed, the multiple correspondence analysis was implemented for the second-year responses (November 2021). DASS21 scores of our three variables stress, anxiety, and depression, were distributed in three grades: normal, mild to severe, and extremely severe (Table 5). From a total of 2916 students, 13 participants were removed. A total of 2903 students participated in the multiple correspondence analysis.

### 3.3. Multiple Correlations and Statistical Analyses

The multiple statistical analysis was assessed on the prevalence of stress, anxiety, and depression (from November 2021), in association with the participants’ demographic characteristics. Therefore, the Burt Tables were produced, alongside with the low-dimensional Euclidean spaces from which derived all the correlations (Table 6).

As shown (Table 6), there were significant differences on the anxiety, stress, and depression levels and several sociodemographic and psychological features of the students. Younger students, female, and unmarried participants presented significantly increased levels of our three variables, in comparison to older students, males, and married respondents (*p*-Value < 0.00001). Similarly, all three variables were elevated for students who claimed to have received psychological or psychiatric treatment and those who were receiving psychotropic drugs during the surveys’ launch periods (*p*-Value < 0.00001). Vaccinated students’ responses revealed an augmentation in anxiety levels, in comparison to the unvaccinated respondents (*p*-Value = 0.0093). Also, concerns for an impending lockdown affected the levels of stress, anxiety, and depression (*p*-Value < 0.00001) (Table 7).

The same differences were also revealed on the gender-based analysis of our three variables regarding the psychological or psychiatric treatment, the psychotropic drugs intake, and the concerns on an impending lockdown (Table 8 and Table 9). Female vaccinated students were significantly more anxious in comparison to the non-vaccinated (*p*-Value = 0.0079). Also, male students who lived alone presented higher anxiety levels in comparison to those who lived with one or more people (*p*-Value = 0.039).

## 4. Discussion

This study assessed the stress, anxiety, and depression levels of the Aristotle University of Thessaloniki community, through the DASS21, during the 2-year ongoing pandemic. Severe and extremely severe prevalence was revealed in alarmingly high rates. The deterioration of students’ mental health was conspicuous in both years. These findings were similar to initial research projects carried out in other Greek universities [17,32,33]. Our study revealed a significant upward pace in students’ stress, anxiety, and depression levels during the 2-year evaluation.

Setting a pre-pandemic background, studies before the COVID-19 outbreak in Greece revealed a mild depression prevalence, but the results were in most cases conflicting [34,35,36,37,38,39,40]. A survey launched in 2015 reported mild to normal depression, related to marital status and previous psychiatric evaluation [34]. During the years 2009–2011, a study assessed the changes in mental health in the general population of Greece, due to the beginning of the economic crisis. Depression levels were increased, while stress remained stable [35]. Moreover, the fact that students, particularly from Greece, are prone to alcohol consumption during their first years of college, could lead to psychological imbalance and negative feelings [36,37]. On the contrary, another pre-pandemic study assessed the effects of the Mediterranean diet on Greek university students and found reduced levels of depression and stress, due to the consumption of specific local products [38]. Regarding mental illness, a pre-pandemic large study in the AUTh found that students claim to be familiar with mental illness, but through unreliable sources [39]. This finding reinforced fear of stigma, especially during the COVID-19 pandemic. Consequently, their imbalanced psychology, the fear of stigma, and further burden due to the daily changes of routine, posed severe danger to the mental wellness of AUTh students [40].

In the current research, approximately 22–26% of the students were integrated to the severe and extreme severe scales of the three study variables (stress, anxiety, and depression). The present 2-year analysis of the AUTh community revealed similarities with the international studies. According to Carr et al., a large percentage of a UK university community was on the verge of depression and severe anxiety disorder, with analogous proportions (30% of the students) [41]. Van Niekerk et al., launched a resemblant extensive survey during the two quarantine periods (2020 and 2021) in an Eastern Cape university and suggested that the risk of mental health deterioration should not be underestimated [42]. In the United Arab Emirates, half of the participants were at psychological distress, with those suffering of mental illness being at highest risk [43]. In China, an early COVID-19 study revealed increased levels of fear, which were associated with the prevalence of depression in students who were close to graduation [9]. Symptoms of depression and anxiety were also found in students during lockdown periods in China, 1 year after the onset of the pandemic [44,45]. Similarly, in Malaysia, a significant prevalence of anxiety in students was observed during the first year of the pandemic [14]. However, their extreme scores were significantly lower than the ones found in the AUTh students. A study conducted in seven united states of America revealed that students with low quality of life and health, of low income, and of young age were at psychological distress due to the pandemic [12]. However, the case of Sweden was different [46]. The DASS21 survey was launched in Swedish students during the first 3 months of the pandemic and no significant increase in stress, anxiety, and depression levels was revealed. On the contrary, Swedish students’ mental state was improved, especially during the summer months of the first year of the pandemic [46].

Furthermore, the increased levels of stress and anxiety were correlated with female gender, between the AUTh students. More than the half of the AUth female participants suffered from mild to severe stress. The numbers of female participants that scored on the extreme stress, anxiety, and depression scales was high (above the expected scores on both launches) (Table 3). The male sample was also affected by the pandemic but not at such extreme scales. Stress and depression were above the expected values for male students during the second launch (Table 4). The higher risk of psychological distress in females was confirmed by our findings, in accordance to other studies [12,13,33]. The present study confirmed that the ongoing pandemic has significantly affected the female population of the AUTh, especially after 2 years of consecutive measures and restrictions.

Several students declared to have received psychological or psychiatric care in the past. There was a significant correlation of the increased psychiatric treatment and the elevated stress, anxiety, or depression levels of the AUTh community. These findings were also in line with evidence from pre-pandemic studies [34]. A balanced social environment and resilience of character are essential for preserving the mental well-being during the home-confinement periods [47].

The Aristotle University of Thessaloniki has established a 24-h-communication line for members who seek counseling and psychological support. Records so far were in alignment with the evidence presented in this study. The cases of the AUTh community members who seek consultation and psychological support has quintupled. Two hundred and fifty members of the university community are currently supported by two psychologists (support center of AUTh) [48]. Therefore, re-evaluation through similar studies by the end of the next years is vital.

### Strengths and Limitations

In the present study, the DASS21 was used independently of any other psychometric scales. This way, the researchers tried to increase the chances of higher participation rates, by constructing a short and coherent questionnaire that could independently screen for stress, anxiety, and depression our sample. Limitations were the lack of evaluation on routine habits (e.g., alcohol consumption, food, etc.), the lack of detailed psychiatric background evaluation of the students, and the lack of specialized questions on the respondents’ physical and mental health. The authors made an effort to avoid long questionnaires in order to minimize the risk of losing participations. Regarding the female students’ significantly higher participation in the survey, it should be noted that the AUTh reports a female population of 54%. The responses here were about 70%, which is much higher. Even though expected based on the literature, the higher female participation should be considered in the interpretation of our findings.

The strengths of this study are the repetition of the analysis, the multiple correlations analysis, and the number of participants.

## 5. Conclusions

The community of Aristotle University of Thessaloniki has been greatly affected by COVID-19, with students presenting high levels of stress, anxiety, and depression during the pandemic. The deterioration of the mental health of AUTh students was in line with international and Greek research data. The analysis of the demographic and social variables indicated a statistically significant correlation between elevated stress, anxiety, and depression levels and gender, age, and psychiatric treatment. Vaccination against COVID-19 was not associated with any significant difference on stress, anxiety, and depression levels. The majority of the female participants were worried about the future and concerned for an impending quarantine confinement. The continuous implementation of restrictive measures poses significant risks to the mental health of students. It is necessary to continue the evaluation in universities and colleges every year, even after the de-escalation of the pandemic.

## Figures and Tables

**Table 1:** The questionnaire used in both years’ surveys.

Α. Basic Information (Demographic, Psychological, COVID-19)		
1. Age range		
2. Sex		
3. Marital Status		
4. Health Professional (YES/NO)		
5. Cohabitation		
6. Changes in professional activity		
7. Test for SARS-CoV-2 (YES/NO)		
8. Known person diagnosed positive for COVID-19 (YES/NO)		
9. Symptoms manifestation		
10. Vaccination against COVID-19 (YES/NO) (only in the second launch, November 2021)		
11. Concerns about an impending lockdown (0, 1, 2, 3) (only in the second launch, November 2021)		
12. Psychological or psychiatric treatment in the past (YES/NO)		
13. Psychological or psychiatric treatment at this time (YES/NO)		
14. Psychotropic drugs intake (YES/NO)		
15. Restriction due to quarantine had positive effects on relationships between people confined within the same house (YES/NO)		
16. Restriction due to quarantine had negative effects on relationships between people confined within the same house (YES/NO)		
17. Restriction due to quarantine had positive effects on social relations		
18. Restriction due to quarantine had negative effects on social relations		
**Β. Information about the University Status**		
19. Academic capacity (Students, Administrative staff, Academic staff)		
20. Category of students (Undergraduate BSc or MD, MSc, PhD)		
21. Year of study (for undergraduate students)		
**C. DASS21 (Likert-4 Scale)**		
22. I found it hard to wind down (s)	29. I felt that I was using a lot of nervous energy (s)	36. I felt I was close to panic (a)
23. I was aware of dryness of my mouth (a)	30. I was worried about situations in which I might panic and make a fool of myself (a)	37. I was unable to become enthusiastic about anything (d)
24. I couldn’t seem to experience any positive feeling at all (d)	31. I felt that I had nothing to look forward to (d)	38. I felt I wasn’t worth much as a person (d)
25. I experienced breathing difficulty (e.g., excessively rapid breathing, breathlessness in the absence of physical exertion) (a)	32. I found myself getting agitated (s)	39. I felt that I was rather touchy (s)
26. I found it difficult to work up the initiative to do things (d)	33. I found it difficult to relax (s)	40. I was aware of the action of my heart in the absence of physical exertion (e.g., sense of heart rate increase, heart missing a beat) (a)
27. I tended to over-react to situations (s)	34. I felt down-hearted and blue (d)	41. I felt scared without any good reason (a)
28. I experienced trembling (e.g., in the hands) (a)	35. I was intolerant of anything that kept me from getting on with what I was doing (s)	42. I felt that life was meaningless (d)

(a): evaluation of anxiety, (s): evaluation of stress, (d): evaluation of depression.

**Table 2:** DASS21 results of the Aristotle University of Thessaloniki students during the 2 years of the pandemic (2020–2021). The comparisons were performed with the chi-square test (*p*-Value significant at 0.05).

Students’ Scores *(%)*	Stress (%)		Anxiety (%)		Depression (%)	
	2020	2021	2020	2021	2020	2021
Normal	1059 *(50.2)*	1188 *(40.7)*	1265 *(60.0)*	1295 *(44.4)*	844 *(40.0)*	1008 *(34.6)*
Mild	265 *(12.6)*	347 *(11.9)*	271 *(12.8)*	421 *(14.4)*	275 *(13.0)*	303 *(10.4)*
Moderate	316 *(15.0)*	476 *(16.3)*	194 *(9.2)*	298 *(10.2)*	433 *(20.5)*	590 *(20.2)*
Severe	294 *(13.9)*	502 *(17.2)*	116 *(5.5)*	243 *(8.3)*	219 *(10.4)*	382 *(13.1)*
Extreme severe	176 *(8.3)*	403 *(13.8)*	264 *(12.5)*	659 *(22.6)*	339 *(16.1)*	633 *(21.7)*
Total	2110 *(100.0)*	2916 *(100.0)*	2110 *(100.0)*	2916 *(100.0)*	2110 *(100.0)*	2916 *(100.0)*
*p*-Values	<0.00001		<0.00001		<0.00001	

**Table 3:** Chi-square statistical analysis between female students during the 2 years, based on the scores of the DASS21 scale (*p*-Value significant at 0.05).

Female Students	Stress (%)		Anxiety (%)		Depression (%)	
	2020	2021	2020	2021	2020	2021
Normal	718 (45.8)	736 (35.9)	890 (56.7)	817 (39.8)	584 (37.2)	662 (32.3)
Mild	207 (13.2)	252 (12.3)	203 (12.9)	288 (14.0)	194 (12.4)	197 (9.6)
Moderate	252 (16.1)	360 (17.6)	151 (9.6)	239 (11.7)	331 (21.2)	424 (20.7)
Severe	239 (15.2)	389 (18.9)	97 (6.4)	181 (8.8)	181 (11.5)	283 (13.8)
Extreme severe	152 (9.7)	314 (15.3)	227 (14.4)	526 (25.6)	278 (17.7)	485 (23.6)
*p*-Values	<0.00001		<0.00001		<0.00001	

**Table 4:** Chi-square statistical analysis between male students during the 2 years, based on the scores of the DASS21 scale (*p*-Value significant at 0.05).

Male Students	Stress (%)		Anxiety (%)		Depression (%)	
	2020	2021	2020	2021	2020	2021
Normal	341 (62.9)	452 (52.2)	375 (69.2)	482 (55.7)	260 (48.0)	341 (39.4)
Mild	58 (10.7)	95 (11.0)	68 (12.5)	129 (14.9)	81 (14.9)	107 (12.4)
Moderate	64 (11.7)	116 (13.4)	43 (7.9)	61 (7.0)	102 (18.8)	164 (19.0)
Severe	55 (10.1)	113 (13.1)	19 (3.5)	60 (6.9)	38 (7.0)	98 (11.3)
Extreme severe	24 (4.4)	89 (10.3)	37 (6.8)	133 (15.5)	61 (11.3)	155 (17.9)
*p*-Values	0.00008		<0.00001		0.00010	

**Table 5:** The percentages of the three distribution grades for all participants.

	DASS21 Score	Normal (%)	Mild to Severe (%)	Extreme Severe (%)
Students (N = 2903)	Stress	651 (22.4)	1501 (51.7)	751 (25.9)
	Anxiety	1290 (44.4)	958 (33.0)	655 (22.6)
	Depression	1003 (34.6)	1269 (43.7)	631 (21.7)

**Table 6:** The Burt table based on the variables and students’ responses and statistical analysis. The variables are presented in odds ratios (ORs).

		Stress			Anxiety			Depression		
Burt Table/ Odds-Ratios		Normal	Mild to Severe	Extreme Severe	Normal	Mild to Severe	Extreme Severe	Normal	Mild to Severe	Extreme Severe
Age range	18–25	0.26	1.09	0.37	0.72	0.51	0.32	0.47	0.82	0.30
	26–35	0.30	1.08	0.34	0.96	0.46	0.24	0.62	0.72	0.25
	36–45	0.62	0.88	0.17	1.67	0.38	0.11	1.21	0.51	0.13
	≥46	0.71	0.82	0.16	2.07	0.31	0.10	1.41	0.46	0.11
	*p*-Values	<0.00001			<0.00001			<0.00001		
Gender	Male	0.45	0.97	0.24	1.24	0.42	0.18	0.65	0.74	0.22
	Female	0.23	1.12	0.40	0.66	0.53	0.34	0.48	0.79	0.30
	*p*-Values	<0.00001			<0.00001			0.00022		
Marital status	Unmarried	0.27	1.08	0.36	0.76	0.50	0.30	0.50	0.80	0.29
	Other	0.61	0.91	0.17	1.69	0.34	0.13	1.29	0.46	0.14
	*p*-Values	<0.00001			<0.00001			<0.00001		
Cohabitation status	I live alone	0.27	1.09	0.36	0.74	0.49	0.33	0.49	0.82	0.29
	With 1 person	0.28	1.08	0.35	0.77	0.50	0.30	0.50	0.76	0.30
	With 2 or more	0.31	1.05	0.34	0.87	0.49	0.26	0.58	0.76	0.26
	*p*-Values	0.79			0.12			0.26		
Vaccinated	Yes	0.28	1.07	0.36	0.77	0.49	0.31	0.52	0.78	0.29
	No	0.33	1.05	0.32	0.96	0.50	0.22	0.58	0.77	0.25
	*p*-Values	0.28			0.0093			0.36		
Concerns about an impending lockdown	None	0.57	0.78	0.25	1.22	0.41	0.19	0.76	0.63	0.22
	Little	0.38	1.15	0.23	1.10	0.46	0.19	0.69	0.75	0.19
	Much	0.23	1.25	0.35	0.69	0.55	0.30	0.48	0.85	0.28
	Very Much	0.18	0.91	0.59	0.53	0.48	0.49	0.35	0.77	0.44
	*p*-Values	<0.00001			<0.00001			<0.00001		
Psychological or psychiatric treatment in the past	Yes	0.15	1.08	0.54	0.47	0.55	0.48	0.29	0.86	0.45
	No	0.34	1.07	0.29	0.95	0.47	0.24	0.63	0.75	0.23
	*p*-Values	<0.00001			<0.00001			<0.00001		
Currently taking psychotropic drugs	Yes	0.14	0.66	0.93	0.27	0.54	0.77	0.23	0.61	0.77
	No	0.30	1.09	0.33	0.83	0.49	0.28	0.54	0.78	0.26
	*p*-Values	<0.00001			<0.00001			<0.00001		

*p*-Value significant at 0.05.

**Table 7:** The severe extreme stress anxiety depression odds ratios (ORs).

Extreme Severe Scale	Stress	Anxiety	Depression
Age range: 18–25 vs. 26–35	1.1	1.33	1.2
Gender: Female vs. Male	1.7	1.9	1.4
Marital status: Unmarried vs. Other	2.1	2.3	2.1
Cohabitation status:			
I live alone vs. Live With 1 person	1.0	1.1	1.0
I live alone vs. With 2 or more	1.1	1.3	1.1
Vaccinated: Yes vs. No	1.1	1.4	1.2
Concerns about an impending lockdown:			
None vs. Little	1.1	1.0	1.1
None vs. Much	0.7	0.6	0.8
None vs. Very Much	0.4	0.4	0.5
Psychological or psychiatric treatment in the past:			
Yes vs. No	1.9	2.0	2.0
Currently taking psychotropic drugs:			
Yes vs. No	2.8	2.8	3.0

**Table 8:** Gender-based Burt table of the male students and statistical analysis (*p*-Values significant at 0.05). The variables are presented in odds ratios (ORs).

Burt Table/ Odds Ratios		Male Students								
		Stress			Anxiety			Depression		
		Normal	Mild to Severe	Extreme Severe	Normal	Mild to Severe	Extreme Severe	Normal	Mild to Severe	Extreme Severe
Marital status	Unmarried	0.44	0.99	0.25	1.20	0.42	0.19	0.61	0.77	0.23
	Other	0.84	0.70	0.15	2.29	0.35	0.05	2.07	0.39	0.05
	*p*-Values	0.085			0.056			0.0002		
Cohabitation status	I live alone	0.45	0.92	0.26	1.15	0.40	0.22	0.61	0.74	0.24
	With 1 person	0.39	1.00	0.28	1.00	0.45	0.24	0.53	0.78	0.27
	With 2 or more	0.49	0.99	0.21	1.46	0.41	0.13	0.75	0.72	0.18
	*p*-Values	0.64			0.039			0.22		
Vaccinated	Yes	0.43	1.00	0.25	1.20	0.43	0.19	0.64	0.75	0.22
	No	0.54	0.85	0.23	1.45	0.37	0.16	0.71	0.69	0.21
	*p*-Values	0.46			0.57			0.83		
Concerns about an impending lockdown	None	0.79	0.70	0.17	1.57	0.36	0.14	0.82	0.62	0.20
	Little	0.61	0.80	0.21	1.65	0.38	0.12	0.81	0.76	0.14
	Much	0.33	1.44	0.19	1.06	0.52	0.17	0.60	0.81	0.21
	Very Much	0.26	0.93	0.45	0.87	0.38	0.35	0.43	0.72	0.39
	*p*-Values	<0.00001			0.00064			0.0004		
Psychological or psychiatric treatment in the past	Yes	0.21	1.09	0.43	0.65	0.56	0.33	0.26	1.07	0.38
	No	0.53	0.94	0.20	1.47	0.38	0.15	0.80	0.67	0.18
	*p*-Values	<0.00001			<0.00001			<0.00001		
Currently taking psychotropic drugs	Yes	0.26	0.61	0.71	0.38	0.53	0.61	0.26	0.45	0.93
	No	0.46	0.98	0.23	1.29	0.41	0.17	0.67	0.75	0.20
	*p*-Values	0.01			0.00072			0.00007		

**Table 9:** Gender-based Burt table of the female students and statistical analysis (*p*-Values significant at 0.05). The variables are presented in odds ratios (ORs).

Burt Table/ Odds Ratios		Female Students								
		Stress			Anxiety			Depression		
		Normal	Mild to Severe	Extreme Severe	Normal	Mild to Severe	Extreme Severe	Normal	Mild to Severe	Extreme Severe
Marital status	Unmarried	0.21	1.13	0.42	0.63	0.54	0.36	0.45	0.82	0.31
	Other	0.54	1.00	0.18	1.52	0.34	0.17	1.10	0.48	0.18
	*p*-Values	<0.00001			<0.0001			<0.00001		
Cohabitation status	I live alone	0.21	1.17	0.41	0.61	0.53	0.38	0.44	0.85	0.31
	With 1 person	0.24	1.11	0.39	0.69	0.52	0.33	0.49	0.75	0.32
	With 2 or more	0.24	1.08	0.40	0.69	0.53	0.32	0.51	0.77	0.29
	*p*-Values	0.83			0.60			0.68		
Vaccinated	Yes	0.22	1.11	0.41	0.63	0.52	0.37	0.47	0.79	0.31
	No	0.26	1.15	0.35	0.81	0.55	0.24	0.53	0.81	0.26
	*p*-Values	0.41			0.0079			0.34		
Concerns about an impending lockdown	None	0.45	0.84	0.31	1.04	0.45	0.22	0.73	0.65	0.23
	Little	0.29	1.36	0.24	0.91	0.51	0.23	0.63	0.75	0.22
	Much	0.19	1.19	0.41	0.60	0.57	0.36	0.44	0.87	0.30
	Very Much	0.15	0.91	0.65	0.43	0.52	0.55	0.32	0.79	0.46
	*p*-Values	<0.00001			<0.00001			<0.00001		
Psychological or psychiatric treatment in the past	Yes	0.13	1.07	0.58	0.42	0.55	0.54	0.30	0.81	0.48
	No	0.27	1.13	0.34	0.77	0.52	0.29	0.56	0.79	0.25
	*p*-Values	<0.00001			< 0.00001			<0.00001		
Currently taking psychotropic drugs	Yes	0.10	0.68	1.03	0.23	0.55	0.84	0.22	0.68	0.72
	No	0.24	1.14	0.38	0.69	0.53	0.33	0.49	0.80	0.29
	*p*-Values	0.00003			0.00002			0.00013		

## Data Availability

Not applicable.

## Supplementary Materials

The following supporting information can be downloaded at: https://www.mdpi.com/article/10.3390/jcm11154263/s1, Table S1: Demographic characteristics of the participants during the first and second year of completing the questionnaire. Table S2: Questions about COVID-19 during the 2 years, Table S3: Participants’ mental health characteristics and social burden due to the pandemic, Table S4: University status of participants, Table S5: Vaccination against COVID-19 infection (second-year participants only), Table S6: Concern about impending lockdown (second year participants), Table S7: Study of gender correlation and basic questions during the first year of completing the questionnaire (2020), Table S8: Gender-based student scores during the first year of the survey (2020), Table S9: Study of gender correlation and basic questions during the second year of completing the questionnaire (2021), Table S10: Gender-based student scores during the second year of the survey (2021), Table S11: Do you know anyone who has been diagnosed with COVID-19? (2020), Table S12: Do you know anyone who has been diagnosed with COVID-19? (2021), Table S13: Correlation of the question “Do you know anyone who has been diagnosed with COVID-19 infection?” with the DASS21 score during the 1st year of the survey, Table S14. Correlation of the question “Do you know anyone who has been diagnosed with COVID-19 infection?” with the score DASS21 during the second year of the research.
