# Supplementary material for: Stress, Anxiety and Depression Prevalence among Greek University Students during COVID-19 Pandemic: A Two-Year Survey
Source: J Clin Med. 2022 Jul 22;11(15):4263. doi: 10.3390/jcm11154263 (PMC9332455; doi:10.3390/jcm11154263)
Supplement: Supplementary file 1 [file jcm-11-04263-s001.zip › jcm-1800342-supplementary.pdf]

Table S1. Demographic characteristics of the participants during the 1st and 2nd year of completing the questionnaire.

Characteristics	Participants on 2020 (%)	Participants on 2021 (%)
**Age**		
18-25	1719 (74.0)	2369 (75.0)
26-35	226 (9.7)	339 (10.7)
36-45	143 (6.2)	170 (5.4)
46-55	150 (6.5)	187 (5.9)
> = 56	84 (3.6)	95 (3.0)
**Sex**		
Female	1694 (73.0)	2205 (69.8)
Male	628 (27.0)	955 (30.2)
**Marital Status**		
Single	1971 (84.9)	2781 (88.0)
Married or with partner	304 (13.1)	324 (10.3)
Divorced or separated	41 (1.7)	51 (1.6)
Widowed	6 (0.3)	4 (0.1)
**Health worker**		
Yes	202 (8.7)	244 (7.7)
No	2120 (91.3)	2916 (92.3)
**Live with**		
Alone	340 (14.6)	983 (31.1)
1 person	477 (20.5)	799 (25.3)
2-4 people	1336 (57.5)	1252 (39.6)
5 or more people	169 (7.4)	126 (4.0)
**Changes in employment activity**		
Yes	756 (32.6)	870 (27.5)
No	399 (17.1)	677 (21.5)
No employment activity	1167 (50.3)	1613 (51.0)
**Total**	2322 (100.0)	3160 (100.0)

Table S2. Questions about Covid-19 during the two years.

Characteristics	Participants on 2020 (%)	Participants on 2021 (%)
**Tested for COVID-19**		
Yes	1691 (72.8)	2872 (90.9)
No	631 (27.2)	288 (9.1)
**Know patient with COVID-19 diagnosis**		
Yes	2033 (87.6)	2984 (94.4)
No	289 (12.4)	176 (5.6)
SUM	2322 (100.0)	3160 (100.0)
**If YES, Reported COVID-19 symptoms**		
No Symptoms	117 (5.8)	87 (2.9)
Mild	896 (44.1)	1007 (33.7)
Moderate	647 (31.8)	1202 (40.3)
Severe	254 (12.4)	420 (14.1)
Death	119 (5.9)	268 (9.0)
**Total of those reported YES**	2033 (100.0)	2984 (100.0)

Table S3. Participants' mental health characteristics and social burden due to the pandemic.

Characteristics	Participants on 2020 (%)	Participants on 2021 (%)
**Previous psychological or psychiatric treatment**		
Yes	439 (18.9)	796 (25.2)
No	1883 (81.1)	2364 (74.8)
**Current psychological or psychiatric treatment**		
Yes	159 (6.8)	458 (14.5)
No	2163 (93.2)	2702 (85.5)
**Current intake of psychoactive medication**		
Yes	48 (2.1)	122 (3.9)
No	2274 (97.9)	3038 (96.1)
**Positive effects of confinement on relationships with confined people**		
Yes	1195 (51.5)	1474 (46.6)
No	1127 (48.5)	1686 (53.4)
**Negative effects of confinement on relationships with confined people**		
Yes	905 (39.0)	1566 (49.6)
No	1417 (61.0)	1594 (50.4)
**Positive effects on social relationships**		
Yes	298 (12.8)	521 (16.5)
No	2024 (87.2)	2639 (83.5)
**Negative effects on social relationships**		
Yes	1782 (76.7)	2502 (79.2)
No	540 (23.3)	658 (20.8)
**Total**	2322 (100.0)	3160 (100.0)

Table S4. University status of participants.

University Situation	Participants on 2020 (%)	Participants on 2021 (%)
**Group**		
Student	2110 (90.9)	2916 (92.2)
Administrative staff	86 (3.7)	106 (3.4)
Faculty members and academic staff	126 (5.4)	138 (4.4)
**Total**	2322 (100.0)	3160 (100.0)
**Student group**		
Undergraduate	1724 (81.7)	2394 (82.1)
Master	286 (13.6)	385 (13.2)
PhD	100 (4.7)	137 (4.7)
**Total of Students**	2110 (100.0)	2916 (100.0)
**Year (undergraduate students)**		
1	599 (34.7)	644 (26.9)
2	298 (17.3)	416 (17.4)
3	281 (16.3)	399 (16.7)
4	208 (12.1)	400 (16.7)
5	143 (8.3)	231 (9.6)
6	102 (5.9)	146 (6.1)
Preferred not to say it	93 (5.4)	158 (6.6)
**Total of undergraduate Students**	1724 (100.0)	2394 (100.0)

Table S5. Vaccination against Covid-19 infection (2nd year participants only)

Vaccinated:	YES	NO	TOTAL	p-values (chi-squared)
**Sex**				
Female	1812	393	2205	.711
Male	790	165	955	
**Health worker**				
Yes	209	35	244	.157
No	2393	523	2916	
**Group**				
Student	2373	543	2916	**<.00001**
Administrative staff	99	7	106	
Faculty members and academic staff	130	8	138	
**Total**	**2602**	**558**	**3160**	
**Student group (N = 2916)**				
Undergraduate	1923	471	2394	**.00098**
Master	324	61	385	
PhD	126	11	137	
**Total**	**2373**	**543**	**2916**	

Table S6. Concern about impending lockdown (second year participants)

**Worries about lockdown:**	**NOT AT ALL**	**A LITTLE**	**MUCH**	**VERY MUCH**	**SUM**	**p-values (chi-squared)**
**Sex**						
Female	228	640	795	542	2205	**<.00001**
Male	147	310	292	206	955	
**Health worker**						
Yes	28	73	90	53	244	.816
No	347	877	997	695	2916	
**University status**						
Student	335	862	1019	700	2916	**.024**
Administrative staff	17	34	33	22	106	
Faculty members and academic staff	23	54	35	26	138	
**Total**	**375**	**950**	**1087**	**748**	**3160**	

Table S7. Study of gender correlation and basic questions during the 1st year of completing the questionnaire (2020).

Student Gender / Questions	Female	Male	Total	p-values (chi-squared)
**Psychological or psychiatric treatment at this time?**				
Yes	126	23	149	**.003**
No	1442	519	1961	
**Are you taking psychotropic drugs?**				
Yes	34	9	43	.470
No	1534	533	2067	
**Did confinement have a positive effect on relationships within the same house?**				
Yes	826	256	1082	**.029**
No	742	286	1028	
**Did confinement have positive effects on social relations?**				
Yes	205	66	271	.590
No	1363	476	1839	
**Total**	**1568**	**542**	**2110**	

Table S8. Gender-based student scores during the 1st year of the survey (2020).

Students (November 2020)	Stress (%)		Anxiety (%)		Depression (%)	
	Male	Female	Male	Female	Male	Female
**Normal**	341 ***(62.9)***	718 ***(45.8)***	375 ***(69.3)***	890 ***(56.7)***	260 ***(48.0)***	584 ***(37.3)***
**Mild**	58 ***(10.7)***	207 ***(13.2)***	68 ***(12.5)***	203 ***(12.9)***	81 ***(14.9)***	194 ***(12.4)***
**Moderate**	64 ***(11.8)***	252 ***(16.1)***	43 ***(7.9)***	151 ***(9.5)***	102 ***(18.8)***	331 ***(21.1)***
**Severe**	55 ***(10.2)***	239 ***(15.2)***	19 ***(3.5)***	97 ***(6.2)***	38 ***(7.1)***	181 ***(11.5)***
**Extreme Severe**	24 ***(4.4)***	152 ***(9.7)***	37 ***(6.8)***	227 ***(14.7)***	61 ***(11.2)***	278 ***(17.7)***
**Total**	542 ***(100.0)***	1568 ***(100.0)***	542 ***(100.0)***	1568 ***(100.0)***	542 ***(100.0)***	1568 ***(100.0)***

Table S9. Study of gender correlation and basic questions during the 2nd year of completing the questionnaire (2021).

**Student Gender / Questions**	**Female**	**Male**	**Total**	**p-values (chi-squared)**
**Psychological or psychiatric treatment?**				
Yes	339	96	435	**.00017**
No	1712	769	2481	
**Are you taking psychotropic drugs?**				
Yes	80	29	109	.476
No	1971	836	2807	
**Did confinement have a positive effect on relationships within the same house?**				
Yes	991	358	1349	**.00061**
No	1060	507	1567	
**Did confinement have positive effects on social relations?**				
Yes	330	150	480	.405
No	1721	715	2436	
**Were you vaccinated against Covid-19 (November 2021)?**				
Yes	1664	705	2369	.814
No	387	160	547	
**Total**	**2051**	**865**	**2916**	

Table S10. Gender-based student scores during the 2nd year of the survey (2021).

Students (November 2021)	Stress (%)		Anxiety (%)		Depression (%)	
	Male	Female	Male	Female	Male	Female
**Normal**	452 ***(52.3)***	736 ***(35.9)***	482 ***(69.3)***	817 ***(39.8)***	341 ***(48.0)***	662 ***(32.3)***
**Mild**	95 ***(11.0)***	252 ***(12.2)***	129 ***(12.5)***	288 ***(14.1)***	107 ***(14.9)***	197 ***(9.6)***
**Moderate**	116 ***(13.4)***	360 ***(17.6)***	61 ***(7.9)***	239 ***(11.7)***	164 ***(18.8)***	424 ***(20.7)***
**Severe**	113 ***(13.0)***	389 ***(19.0)***	60 ***(3.5)***	181 ***(8.8)***	98 ***(7.1)***	283 ***(13.7)***
**Extreme Severe**	89 ***(10.3)***	314 ***(15.3)***	133 ***(6.8)***	526 ***(25.6)***	155 ***(11.2)***	485 ***(23.7)***
**Total**	865 ***(100.0)***	2051 ***(100.0)***	865 ***(100.0)***	2051 ***(100.0)***	865 ***(100.0)***	2051 ***(100.0)***

Table S11. Do you know anyone who has been diagnosed with Covid-19? (2020).

Do you know anyone who has been diagnosed with Covid-19 infection?	Yes	No	Total	p-values (chi-squared)
Psychological or psychiatric treatment?				
Yes	128	21	149	.520
No	1720	241	1961	
Are you taking psychotropic drugs?				
Yes	34	9	43	.087
No	1814	253	2067	
**Total participants of the 1st year**	**1848**	**262**	**2110**	

Table S12. Do you know anyone who has been diagnosed with Covid-19? (2021).

Do you know anyone who has been diagnosed with Covid-19 infection?	Yes	No	Total	p-values (chi-squared)
Psychological or psychiatric treatment?				
Yes	414	21	435	.034
No	2333	148	2481	
Are you taking psychotropic drugs?				
Yes	96	13	103	**.005**
No	2651	156	2807	
Have you been vaccinated?				
Yes	2238	131	2369	.200
No	509	38	547	
**Total 2nd year participants**	**2747**	**169**	**2916**	

Table S13. Correlation of the question "Do you know anyone who has been diagnosed with Covid-19 infection?" with the DASS21 score during the 1st year of the survey.

Students (November 2020)	Stress (%)		Anxiety (%)		Depression (%)	
	Yes	No	Yes	No	Yes	No
**Normal**	915 ***(49.5)***	144 ***(55.0)***	1110 ***(60.0)***	155 ***(59.2)***	742 ***(40.2)***	102 ***(38.8)***
**Mild**	240 ***(13.0)***	25 ***(9.5)***	239 ***(13.0)***	32 ***(12.2)***	242 ***(13.1)***	33 ***(13.0)***
**Moderate**	279 ***(15.1)***	37 ***(14.1)***	162 ***(8.8)***	32 ***(12.2)***	389 ***(21.0)***	44 ***(16.7)***
**Severe**	261 ***(14.1)***	33 ***(12.6)***	100 ***(5.4)***	16 ***(6.1)***	182 ***(9.8)***	37 ***(14.0)***
**Extreme Severe**	153 ***(8.3)***	23 ***(8.8)***	237 ***(12.8)***	27 ***(10.3)***	293 ***(15.9)***	46 ***(17.5)***
**Total**	1848 ***(100.0)***	262 ***(100.0)***	1848 ***(100.0)***	262 ***(100.0)***	1848 ***(100.0)***	262 ***(100.0)***
**P-Values**	.400		.380		.160	

Table S14. Correlation of the question "Do you know anyone who has been diagnosed with Covid-19 infection?" with the score DASS21 during the 2nd year of the research.

Students (November 2021)	Stress (%)		Anxiety (%)		Depression (%)	
	Yes	No	Yes	No	Yes	No
**Normal**	1106 ***(40.2)***	82 ***(48.5)***	1213 ***(44.1)***	82 ***(48.5)***	942 ***(34.2)***	66 ***(39.1)***
**Mild**	334 ***(12.2)***	13 ***(7.7)***	398 ***(14.5)***	23 ***(13.6)***	289 ***(10.5)***	14 ***(8.3)***
**Moderate**	449 ***(16.3)***	27 ***(16.0)***	290 ***(10.6)***	8 ***(4.7)***	557 ***(20.3)***	33 ***(19.5)***
**Severe**	477 ***(17.4)***	25 ***(14.8)***	226 ***(8.2)***	17 ***(10.1)***	367 ***(13.4)***	15 ***(8.8)***
**Extreme Severe**	381 ***(13.9)***	22 ***(13.0)***	620 ***(22.6)***	39 ***(23.1)***	592 ***(21.6)***	41 ***(24.3)***
**Total**	2747 ***(100.0)***	169 ***(100.0)***	2747 ***(100.0)***	169 ***(100.0)***	2747 ***(100.0)***	169 ***(100.0)***
**P-Values**	.230		.150		.300	
